# Contrasting species and functional beta diversity in montane ant assemblages

**DOI:** 10.1111/jbi.12537

**Published:** 2015-05-16

**Authors:** Tom R. Bishop, Mark P. Robertson, Berndt J. van Rensburg, Catherine L. Parr

**Affiliations:** ^1^ Department of Earth, Ocean and Ecological SciencesUniversity of LiverpoolLiverpoolL69 3GPUK; ^2^ Department of Zoology and EntomologyCentre for Invasion BiologyUniversity of PretoriaPretoria0002South Africa; ^3^ School of Biological SciencesUniversity of QueenslandSt LuciaQueensland4072Australia

**Keywords:** Ants, beta diversity, biodiversity, elevational gradient, Formicidae, functional beta diversity, functional traits, nestedness, southern Africa, turnover

## Abstract

**Aim:**

Beta diversity describes the variation in species composition between sites and can be used to infer why different species occupy different parts of the globe. It can be viewed in a number of ways. First, it can be partitioned into two distinct patterns: turnover and nestedness. Second, it can be investigated from either a species identity or a functional‐trait point of view. We aim to document for the first time how these two aspects of beta diversity vary in response to a large environmental gradient.

**Location:**

Maloti‐Drakensberg Mountains, southern Africa.

**Methods:**

We sampled ant assemblages along an extensive elevational gradient (900–3000 m a.s.l.) twice yearly for 7 years, and collected functional‐trait information related to the species’ dietary and habitat‐structure preferences. We used recently developed methods to partition species and functional beta diversity into their turnover and nestedness components. A series of null models were used to test whether the observed beta diversity patterns differed from random expectations.

**Results:**

Species beta diversity was driven by turnover, but functional beta diversity was composed of both turnover and nestedness patterns at different parts of the gradient. Null models revealed that deterministic processes were likely to be responsible for the species patterns but that the functional changes were indistinguishable from stochasticity.

**Main conclusions:**

Different ant species are found with increasing elevation, but they tend to represent an increasingly nested subset of the available functional strategies. This finding is unique and narrows down the list of possible factors that control ant existence across elevation. We conclude that diet and habitat preferences have little role in structuring ant assemblages in montane environments and that some other factor must be driving the non‐random patterns of species turnover. This finding also highlights the importance of distinguishing between different kinds of beta diversity.

## Introduction

The concept of beta diversity has a long history in ecology and can be broadly understood as a measure of the variation in species composition between sites. Beta diversity was originally conceived in order to bridge the gap between local (alpha) and regional (gamma) measures of diversity (Whittaker, 1960) and has since become a multifaceted concept with a large number of verbal and mathematical definitions (Tuomisto, 2010; Anderson *et al*., 2011). Studies of beta diversity describe the extent of compositional differences between sites and also attempt to reveal the assembly mechanisms that drive these differences. Understanding how communities are assembled and structured in space and time, and the variation therein, has basic and applied relevance in ecology (Kraft *et al*., 2011; Beaudrot *et al*., 2013) and conservation science (Paknia & Pfeiffer, 2011; Olivier & van Aarde, 2014). Typically, data on species occurrences at sites across a landscape are used to calculate some estimate of beta diversity, but a number of conceptual advances indicate that this approach may only give us a limited insight into the patterns and drivers of beta diversity.

The first of these advances is the partitioning of beta diversity into separate, antithetical components: turnover and nestedness patterns. Although the distinction between these two components is not new (Harrison *et al*., 1992; Wright & Reeves, 1992; Williams *et al*., 1999), frameworks in which to study them explicitly have only recently been developed (Baselga, 2010; Schmera & Podani, 2011; Carvalho *et al*., 2012). Turnover occurs when existing species are replaced by different ones at new sites, whereas nestedness patterns result when species loss or gain causes species‐poor sites to resemble a strict subset of species‐rich sites (Gaston & Blackburn, 2000). Crucially, these two phenomena imply the operation of different ecological processes. Patterns of turnover are expected to be produced by factors that promote endemism at various spatial scales (Bond *et al*., 2001; Baselga, 2010), whereas nestedness is a result of ordered extinctions or colonizations along gradients (Ulrich *et al*., 2009). Given the variety of underlying mechanisms that can produce turnover and nestedness, it is important that we are able to distinguish between these patterns if we are to fully understand and apply our knowledge of beta diversity (Williams *et al*., 1999; Baselga, 2010; Marini *et al*., 2013).

The second advance is the continued development and implementation of functional‐trait‐based ecology. Species identities alone do not provide information on their ecology and so analyses that only use taxonomic data are inherently limited (McGill *et al*., 2006). By incorporating data on functional traits (measurable aspects of organisms that influence their ecology and performance; McGill *et al*., 2006), we can gain a more detailed insight into biodiversity patterns and processes (Fukami *et al*., 2005; Swenson *et al*., 2012; Villéger *et al*., 2012). In addition, a functional‐trait approach allows comparisons to be made between geographical regions that possess different faunas. Indices of functional alpha diversity are already widely used in the ecological literature (Mouchet *et al*., 2010). More recently, measures of functional beta diversity (Ricotta & Burrascano, 2008; Swenson *et al*., 2011) – and their decomposition into turnover and nestedness components – have been developed (Villéger *et al*., 2013; Cardoso *et al*., 2014).

Here, we explored how the turnover and nestedness components of ant (Hymenoptera: Formicidae) species and functional beta diversity are influenced by elevation. This is the first such investigation of animal beta diversity over an extensive gradient. We characterized functional diversity using a number of morphological measures that relate to the feeding and foraging ecology of the ant species. We hypothesized that these functional traits represent key spectra of ant ecology and could thus drive compositional change across elevations. It must be noted that other behavioural traits that may influence the ecology of species (e.g. foraging time preference or dominance) are not used here: such traits are notoriously difficult to quantify for diverse and little‐studied faunas. In addition, purely morphological approaches have previously been shown to capture a wide range of ecological strategies employed by ants (Weiser & Kaspari, 2006; Bihn *et al*., 2010; Silva & Brandão, 2010).

In conjunction with the morphological trait data, we used an assemblage dataset – sampled twice yearly, representing the two main seasons (wet and dry) – collected over 7 years and ranging in elevation from 900 to 3000 m above sea level (a.s.l.). We asked the following questions: (1) How do species and functional beta diversities relate to changes in elevation? (2) Do these relationships depend on the beta diversity component being analysed or on the season? (3) What can we infer about the ecological processes that drive these patterns? In this case, we are interested in whether deterministic or stochastic processes are in operation. Deterministic processes highlight the role of the niche (e.g. habitat filtering or competitive interactions) in determining the composition of local communities. Stochastic effects, on the other hand, emphasize how random chance generates observed patterns of diversity through sampling and priority effects (Chase & Myers, 2011).

We predicted a distance decay in similarity (increasing beta diversity) with increasing elevational distance. We expected species beta diversity to be driven largely by turnover, because ants (Brühl *et al*., 1999; Longino & Colwell, 2011) and other organisms (Wang *et al*., 2012) typically display elevational turnover patterns (although nestedness is not unknown, e.g. Lessard *et al*., 2007; Bernadou *et al*., 2015). No previous work has looked at the partitioning of functional beta diversity across elevation, but communities can become phylogenetically clustered at high elevations (Machac *et al*., 2011; Hoiss *et al*., 2012; Smith *et al*., 2014). This suggests that functional diversity could also shrink in size and become restricted to particular phenotypes. In addition, functional diversity is known to shrink at higher latitudes and in harsher climates (Stevens *et al*., 2003; Lamanna *et al*., 2014). Consequently, we predict nestedness to underlie our functional beta diversity patterns. We also expected to see strong seasonal effects, based on previous work (Bishop *et al*., 2014) which found that alpha diversity was dependent on season. We predicted that greater beta diversity will be found during the dry season, when conditions become unfavourable for ants, potentially limiting the elevational range of individual species; i.e. elevations will be more dissimilar from each other in the dry season than they are in the wet season.

Our finding that species and functional beta diversity are actually the products of contrasting patterns and processes highlights the need to distinguish between different views of biodiversity and sheds further light on the elevational ecology of ants.

## Materials and methods

### Study site

Sampling took place throughout the Sani Pass, which forms part of the Maloti‐Drakensberg Transfrontier Conservation Area and is classified as part of the grassland biome (Mucina & Rutherford, 2006). Sampling locations were located along an elevational transect ranging from 900 m a.s.l. near the village of Ixopo (30°09′ S; 30°03′ E) to 3000 m a.s.l. at a point above the top of the Sani Pass (29°35′ S; 29°17′ E). Eight sampling locations were established in natural vegetation at 300‐m vertical intervals. For further details, see Bishop *et al*. (2014).

### Data collection

#### Ant sampling

Pitfall traps were used to sample the epigaeic (ground‐dwelling) ant fauna in the wet season (January) and the dry season (September) from 2006 to 2012. Four replicate sampling blocks were established at each elevational site. Blocks were spaced at least 300 m apart. Each block consisted of 10 pitfall traps arranged in two parallel lines with traps 10 m apart. Each trap had a volume of 150 mL, a diameter of 55 mm and a depth of 70 mm. Rain guards were used to prevent flooding. A 50% solution of ethylene glycol was used to preserve the ant specimens that were caught in the traps. Trapping took place over 5 nights in total, but traps were serviced every 2 or 3 days to avoid overfilling. Specimens were transferred into 70% ethanol in the laboratory and identified to morphospecies and species level where possible. These sites and sampling design are the same as those used in Bishop *et al*. (2014).

#### Functional traits

Six morphological traits were measured for each species. These were used in various combinations to produce four indices of ecological importance. The resulting indices are expected to capture ecological variation in the feeding and foraging strategies of the different ant species.

1. Weber's length is a measure of body size taken from the anterodorsal margin of the pronotum to the posteroventral margin of the propodeum (Brown, 1953). Body size can influence prey size selection during solitary foraging (Traniello, 1987). Body size can also influence the microhabitats in which different species forage. Large‐bodied ants are likely to forage in open conditions on the soil surface, whereas smaller species may occupy finer ‘grains’ in closed habitats in the leaf litter and soil (Weiser & Kaspari, 2006; Gibb & Parr, 2013).
2. Eye position is calculated as the interocular distance subtracted from the total head width across the eyes. This measure is divided by Weber's length to control for body size. Large values of eye position indicate dorsally positioned eyes (favoured in open habitats; Gibb & Parr, 2013), whereas small values relate to eyes positioned on the side of the head. This distinction is expected to relate both to habitat complexity and to predatory behaviour. Predatory species tend to have small eyes and this trait is correlated with our measure of eye position (Weiser & Kaspari, 2006).
3. Relative leg length is calculated as the sum of the hind femur length and the hind tibia length, divided by Weber's length. Short relative leg lengths correlate with predatory behaviour (Weiser & Kaspari, 2006). Relative leg length may also relate to the complexity of the habitat occupied. Longer legs can be selected for in simple, planar environments (Gibb & Parr, 2013).
4. Relative mandible length is calculated by dividing the length of the mandible from insertion to tip by the head width across the eyes. This measure expresses the size of the mandible as a proportion of head width. Longer mandibles are expected to relate to specialization in a predatory role (Hölldobler & Wilson, 1990; Gronenberg *et al*., 1997).

Traits were measured to the nearest 0.01 mm using an ocular micrometer attached to a Stemi 2000 stereomicroscope (Carl Zeiss Microscopy, Jena, Germany). Species without eyes were assigned a value of zero for all eye measurements. We used the highest magnification that allowed the structure under measurement to be fitted within the range of the ocular micrometer. Only workers of the minor caste were included in the analyses. Six individuals from each species were measured where possible; 92 species were caught and measured across the entire time series. On average, 5.52 individuals were measured per species.

### Analysis

#### Beta diversity

The species beta‐diversity partition proposed by Baselga (2010) and the analogous partition for functional beta diversity developed by Villéger *et al*. (2013) were used. We chose to use these rather than the alternative developed by Carvalho *et al*. (2012) and Cardoso *et al*. (2014) because we were interested in compositional differences strictly due to nestedness, rather than those due to the more general case of richness differences (Carvalho *et al*., 2012). Differences in richness between elevations have already been investigated at this site (Bishop *et al*., 2014).

For species and functional compositions, three pairwise beta‐diversity metrics were calculated. First, β_sor_ accounts for the total compositional variation between assemblages – including both turnover and nestedness patterns. This is the Sørensen dissimilarity index. Second, β_sim_ captures only compositional changes due to species turnover. This is the Simpson dissimilarity index and is invariant to richness differences (Baselga, 2010). Third, β_sne_ represents the nestedness‐resultant dissimilarity and is calculated as the difference between β_sor_ and β_sim_. For species composition, these pairwise metrics use information on the number of species shared between two sites and the number of species unique to each site. Only species occurrence data were used. For functional composition, the volumes of multivariate trait space shared by two sites and unique to each were used as inputs in the dissimilarity equations (Villéger *et al*., 2013). To generate this multivariate space, a principal coordinates analysis (PCoA) was used to summarize the trait data. The PCoA allows us to break correlations between traits, creating orthogonal ‘traits’. We calculated a species‐by‐species Euclidean distance matrix from scaled and centred trait data. The PCoA was run on this distance matrix and the resulting axes were used as four independent, synthetic traits representing different spectra of ant ecological strategies. The ecological meaning of these axes was interpreted based on the loadings of the raw trait values. Assemblages of ants were projected onto this space as a convex hull, with the synthetic trait values of the present species defining the vertices of the hull (Villéger *et al*., 2008). Species and functional pairwise beta‐diversity measures were calculated using the betapart package in R (Baselga & Orme, 2012; R Core Team, 2013).

#### Observed patterns

For each year and season, the four ant assemblages sampled within each elevational band were pooled in order to create assemblages at the elevational site level. This produced a total of 111 assemblages for analysis (8 elevational sites × 2 seasons × 7 years = 111 assemblages, after one assemblage was removed for having too few species to be projected as a convex hull). The three beta‐diversity metrics (β_sor_, β_sim_ and β_sne_) were then calculated between the lowest‐elevation site (900 m a.s.l.) and the seven higher‐elevation sites. This was carried out for both taxonomic and functional assemblage composition. We limited this analysis to comparisons against the lowest‐elevation site for simplicity and clarity. We present the analyses of all pairwise comparisons in Appendices S1 & S2 in the Supporting Information; the overall finding did not differ. We used generalized linear mixed models (GLMMs) to describe the relationship of each beta‐diversity metric to changes in elevation, and to test whether this depended on the season and type of assemblage composition being used (species or functional composition). A polynomial term of elevation was also included to detect nonlinear patterns. Year was used as a random effect to control for temporal pseudoreplication. The lme4 package in R was used to perform the GLMMs (Bates *et al*., 2014). The numerical variable of change in elevation was centred and standardized to improve the interpretability of the resulting model coefficients (Schielzeth, 2010). An information‐theoretic approach was taken to compare models with different combinations of explanatory variables. Bias‐corrected Akaike information criterion (AIC_c_) values were compared in order to select the best descriptive model for each beta‐diversity metric. Marginal *R* ^2^ (due to fixed effects only) and conditional *R* ^2^ (due to both fixed and random effects) were calculated for each model (Nakagawa & Schielzeth, 2013) using the muMin package in R (Barton, 2013). Model predictions were averaged across years for clarity when plotting. This modelling approach allowed us to simultaneously ask (1) if beta diversity was related to changes in elevation, and (2) whether this relationship differed between species and functional compositions and the seasons.

#### Standardized patterns

To investigate what processes were driving the patterns of beta diversity and to answer our third question, we used a null modelling approach. This tested whether our observed beta‐diversity values were larger or smaller than expected under a stochastic model of community assembly. A separate null modelling procedure was performed for species and functional compositions. For each season and year, a null distribution of beta‐diversity values was generated for each of the three metrics. For species composition, this was carried out by generating 1000 random assemblage matrices using the independent swap algorithm (Gotelli, 2000) and recalculating the beta‐diversity metrics. This algorithm maintains species occurrence frequency and sample species richness while shuffling species co‐occurrence across sites. For functional composition, the assemblage data matrix was kept constant but the synthetic traits associated with each species were randomized 1000 times by randomly shuffling the names of the species in a species‐by‐traits matrix and recalculating the functional beta‐diversity metrics. This procedure retains the structure of the overall trait space, but randomly assigns which species has which phenotype (Swenson, 2014). Standardized effect sizes (SES) were calculated using the observed beta‐diversity values and the mean and standard deviation of the null distributions for species and functional compositions in every year and season:

$$\frac{\text{SES}=\text{observed}-\text{mean (null)}}{\text{SD (null)}}$$

SES values can serve as a measure of departure from a pure null expectation. Values greater than 0 are larger than expected whereas those smaller than 0 are less than expected. Essentially, departures from 0 indicate non‐randomness: values greater than 1.96 or less than −1.96 are significantly greater or less than expected, at α = 0.05. As well as the magnitude of departure from our null expectation, we were also interested in any directional trends in the SES values across the elevational gradient. For this, we used GLMMs as described for the observed beta‐diversity values.

## Results

### Functional trait space

**Figure 1:**
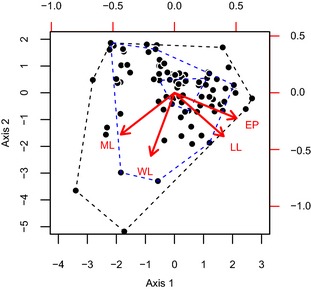
Biplot displaying the structure of the morphological space on the first two principal coordinate axes occupied by the ant fauna of the Sani Pass, southern Africa. Each data point is a species. Lower and left hand axes describe the axis scores (synthetic traits) for each species. Upper and right hand axes describe the loadings of each original trait on the principal coordinate axes. The loadings of each original trait are visualized with red labels and arrows (WL, Weber's length; ML, relative mandible length; LL, relative leg length; EP, eye position). For illustration, black dashed lines represent the convex hull of the entire ant fauna. Inner and outer blue dashed lines represent the convex hull of the assemblages at 3000 and 900 m a.s.l., respectively, for the wet season of 2009. These two assemblages display functional nestedness.

The first two PCoA axes captured *c*. 80% of the variation present in the morphological structure of the ant traits (Table 1). This variation was split nearly evenly between the two axes. Given the loadings of the original traits in the PCoA, we interpret axis 1 as a gradient in predatory specialization (Table 1, Fig. 1): species with traits associated with being predatory specialists (relatively large mandibles, laterally positioned eyes and relatively short legs) had negative scores on axis 1, whereas species with more generalized traits (relatively short mandibles, dorsally positioned eyes and relatively long legs) had positive scores on axis 1. We interpret axis 2 as representing preference for different habitat complexities (Table 1, Fig. 1). Species with traits indicating that they occupy dense, complex habitats (small bodies, relatively short mandibles and short legs) had positive scores on axis 2; species with traits indicating that they occupy open, simple habitats (large bodies, relatively long mandibles and relatively long legs) had negative scores on axis 2. As the first two axes contain most of the variation in the morphological traits, we only use these axes for the interpretation of our results.

**Table 1:** Eigenvalues and trait loadings of a principal coordinates analysis (PCoA) describing the morphological structure of the ant fauna of the Sani Pass, southern Africa. Eigenvalues describe the importance of each PCoA axis in explaining variation in ant traits. Trait loadings indicate how strongly each trait is correlated with each axis and in which direction

	Axis 1	Axis 2	Axis 3	Axis 4
Eigenvalue	154.92	135.82	41.77	31.49
Relative eigenvalue	0.43	0.37	0.11	0.09
Cumulative eigenvalue	0.43	0.80	0.91	1
Trait loadings				
Weber's length	−0.24	−0.69	−0.47	−0.49
Relative leg length	0.63	−0.28	−0.47	0.55
Relative mandible length	−0.54	−0.46	0.29	0.64
Eye position	0.5	−0.48	0.69	−0.23

### Observed patterns

**Figure 2:**
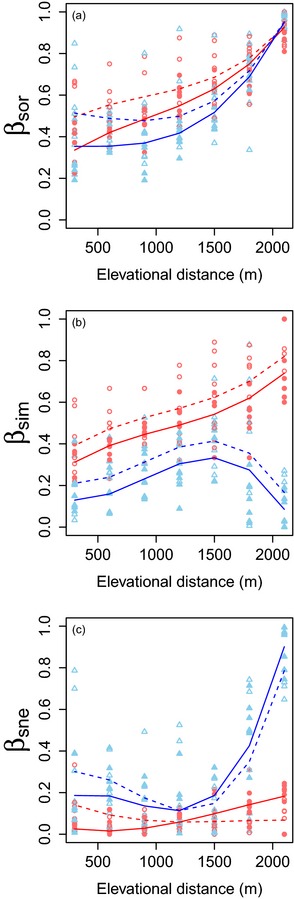
Plots showing the relationship between ant species and functional (a) β_sor_ (total beta diversity), (b) β_sim_ (turnover component), and (c) β_sne_ (nestedness‐resultant component) and elevational distance in the Sani Pass, southern Africa. Red lines and circles indicate species beta diversity. Blue lines and triangles indicate functional beta diversity. Filled shapes and solid lines indicate data and mixed model predictions for the wet season. Empty shapes and dotted lines indicate those for the dry season. Each data point represents a comparison between the lowest elevation (900 m) and the subsequent higher elevations. Data from all years in the dataset are modelled and plotted.

Total species and functional beta diversity (β_sor_) increase with increasing elevational distance (Fig. 2a, Table 2). Total species beta diversity is typically higher than functional beta diversity and is higher during the dry season than during the wet season.

**Table 2:** Model summaries and parameter estimates for generalized linear mixed models explaining variation in observed and standardized beta diversity of ant assemblages within the Sani Pass, southern Africa. The best model, according to the bias‐corrected Akaike information criterion (AIC_c_) is reported. Each column reports results from each metric. β_sor_ is total beta diversity, β_sim_ is turnover and β_sne_ is nestedness. The SES prefix indicates beta diversity values standardized by a null model. Marginal *R* ^2^ (*R* ^2^_m_), measuring variation explained by fixed effects only, and conditional *R* ^2^ (*R* ^2^_c_), measuring variation explained by both fixed and random effects, are given. Estimates are on the standardized scale ± standard error. Blank cells indicate variables not included in the best model for that metric

Model summaries	β_sor_	β_sim_	β_sne_	SES β_sor_	SES β_sim_	SES β_sne_
AIC_c_	−292.42	−224.39	−239.72	492.98	530.79	532.11
*R* ^2^_m_	0.72	0.67	0.73	0.56	0.51	0.5
*R* ^2^_c_	0.76	0.7	0.74	0.56	0.56	0.54
Estimates						
Composition	−0.008 ± 0.01	−0.2 ± 0.01	0.2 ± 0.01	0.7 ± 0.2	−0.09 ± 0.2	0.006 ± 0.2
Season	0.06 ± 0.01	−0.03 ± 0.01	0.09 ± 0.009	−0.2 ± 0.2	0.1 ± 0.1	−0.4 ± 0.2
Elevational distance	0.07 ± 0.01	0.03 ± 0.01	0.04 ± 0.01	0.2 ± 0.1	0.03 ± 0.1	−0.1 ± 0.1
Elevational distance^2^	−0.1 ± 0.008	−0.06 ± 0.009	−0.08 ± 0.007	0.3 ± 0.09	0.5 ± 0.1	−0.4 ± 0.1
Composition: season				0.2 ± 0.2		0.3 ± 0.2
Composition: elevational distance	0.07 ± 0.02	0.003 ± 0.02	0.07 ± 0.01	1 ± 0.2	−1 ± 0.1	2 ± 0.2
Composition: elevational distance^2^	0.02 ± 0.01	0.03 ± 0.01	−0.02 ± 0.009	−0.4 ± 0.1	−0.4 ± 0.1	0.3 ± 0.1
Elevational distance: season		−0.03 ± 0.01	0.03 ± 0.007	0.05 ± 0.2	0.2 ± 0.1	0.05 ± 0.2
Elevational distance: elevational distance^2^	0.08 ± 0.005	0.07 ± 0.006	0.02 ± 0.004			
Elevational distance^2^: season	−0.01 ± 0.007		−0.02 ± 0.006			
Composition: elevational distance: season				−0.6 ± 0.2		−0.6 ± 0.2
Composition: elevational distance: elevational distance^2^	−0.03 ± 0.007	−0.06 ± 0.009	0.03 ± 0.006			

Species and functional turnover (β_sim_) patterns differ in their response to changing elevation (Fig. 2b, Table 2). Species turnover increases almost linearly with increasing elevational distance, whereas functional turnover peaks at intermediate elevational distances before declining, producing a hump‐shaped relationship (Fig. 2b). Both species and functional turnover are higher during the dry season than in the wet season.

Species nestedness effectively shows no change with elevational distance and is very low (typically less than β_sne_ = 0.2, Fig. 2c). Functional nestedness displays a U‐shaped relationship with elevational distance. It marginally declines from low to intermediate distances and then rapidly increases as elevational distance becomes greater than 1500 m. Both compositions display a small seasonal difference: during the dry season, nestedness is greater at small elevational distances whereas, during the wet season, it is greater at large elevational distances.

Models of beta diversity explain a large proportion of the variation in the data (*R* ^2^_m_ = 0.67–0.73; Table 2). None of these three metrics have qualitatively different results when all pairwise comparisons are included (Appendix S2).

### Standardized patterns

**Figure 3:**
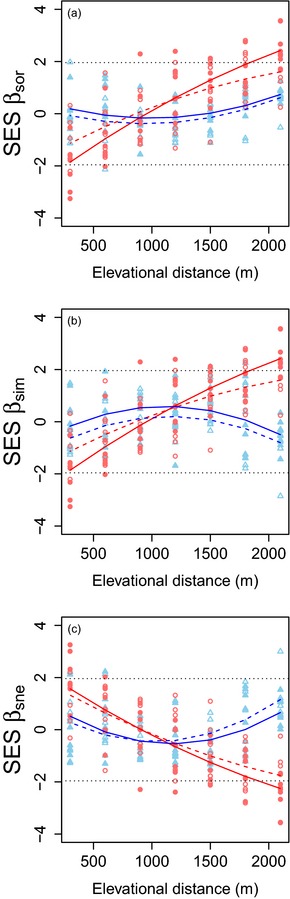
Plots showing the relationship between the standardized effect size (SES) of ant species and functional (a) β_sor_ (total beta diversity), (b) β_sim_ (turnover component) and (c) β_sne_ (nestedness‐resultant component) and elevational distance in the Sani Pass, southern Africa. Red lines and circles indicate species beta diversity. Blue lines and triangles indicate functional beta diversity. Filled shapes and solid lines indicate data and mixed model predictions for the wet season. Empty shapes and dotted lines indicate those for the dry season. Black dotted lines indicate the α = 0.05 threshold of ± 1.96 SES for significantly non‐random values. Each data point represents a comparison between the lowest elevation (900 m) and the subsequent higher elevations. Data from all years in the dataset are modelled and plotted.

The standardized values of total species beta diversity (SES β_sor_) increase with increasing elevational distance (Fig. 3a, Table 2). This increase describes a gradient in SES values from those that are smaller than expected (less than 0), to those that are greater than expected (greater than 0). This relationship has a shallower slope and a higher intercept in the dry season than in the wet season. The standardized values of total functional beta diversity show no strong relationship with elevational distance in either season and deviate little from the null expectation.

There is a near‐identical pattern of results for standardized turnover (SES β_sim_). Standardized species turnover increases from less to greater than expected with increasing elevational distance (Fig. 3b, Table 2). The slope is shallower and the intercept higher in the dry season. Standardized functional turnover displays a slightly hump‐shaped relationship with elevational distance, but again shows no major departure from the null expectation in either season.

Standardized nestedness displays patterns opposite to those for standardized turnover. Standardized species nestedness (SES β_sne_) decreases with increasing elevational distance (Fig. 3c, Table 2) with only a minor change between the seasons. Standardized functional nestedness does not differ from the null expectation but does display a mildly U‐shaped relationship with elevational distance.

All three models explain similar proportions of variation (*R* ^2^_m_ ≈ 0.5; Table 2). These results show that there is a clear trend for species turnover to be lower than expected at small elevational distances and higher than expected at large elevational distances. This pattern is reversed for species nestedness. Neither functional turnover nor nestedness displays any meaningful departure from the null model.

## Discussion

To our knowledge, this is the first study to partition both species and functional beta diversity for animals along an extensive environmental gradient (but see Villéger *et al*., 2013; for an example of wide geographical scope). These results give fresh insight into the mechanisms that may control ant elevational diversity. We find that species compositional change is driven by turnover patterns (Fig. 2) that cannot be properly explained by stochastic effects (Fig. 3). Functional compositional change is more complicated and is produced by a mixture of turnover and nestedness patterns operating between different elevational ranges (Fig. 2). These functional changes, however, appear to be completely random with respect to the underlying species beta diversity (Fig. 3). Consequently, the deterministic changes in ant assemblages across elevation are not a result of the ecological strategies described by the functional traits that we investigate here.

Our broadest finding is that the further apart two sites are, the more dissimilar they are in terms of both species and functional composition (β_sor_; Fig. 2a). We predicted this classic distance decay of assemblage similarity with elevation, and it has been reported for a range of organisms for species and functional traits (Swenson *et al*., 2011; Wang *et al*., 2012). Similar to the results presented by Wang *et al*. (2012) for macroinvertebrates, we find that species beta diversity is driven by turnover. Species tend to specialize at particular bands of elevation rather than exist across the entire gradient. This is in line with our predictions, and similar patterns have been observed for ants in Malaysia (Brühl *et al*., 1999) and Tanzania (Robertson, 2002). These comparable patterns across mountains in tropical and subtropical environments imply that there may be a common underlying mechanism generating ant elevational beta diversity. In addition, the species turnover pattern that we report highlights the importance of mountains as reservoirs of unique biodiversity across their entire range. A different interpretation would be reached if species nestedness was observed – under a nestedness scenario, only the lowest elevations would possess unique species.

We also find that beta diversity tends to be higher in the dry season than in the wet season (Fig. 2, Table 2). We predicted this, because we expected species ranges to shrink into their optimal elevational range during this harsh time of the year, increasing the differences between elevations. This is a relatively small effect, however, and is not consistent across the beta‐diversity components. The amount of nestedness in each season depends on the elevational distance, for example (Fig. 2).

Contrary to our predictions, we see both functional turnover and nestedness. We expected to observe primarily functional nestedness based on previous work which showed that phylogenetic and functional diversity can shrink in harsher climates (Machac *et al*., 2011; Lamanna *et al*., 2014). Our functional turnover patterns show that novel strategies can be favoured in parts of the gradient.

**Figure 4:**
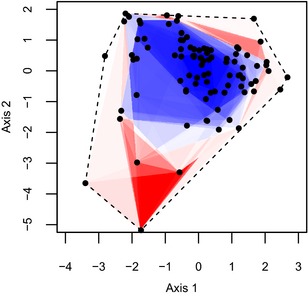
Plot of the first two principal coordinate axes of functional trait spaces occupied by the ant fauna of the Sani Pass, southern Africa. Regions where turnover (darker red) and nestedness (darker blue) dominate are highlighted. The density of turnover and nestedness occurrence throughout the space was calculated separately and then combined to produce a single gradient describing the dominance of either pattern. Turnover was defined as areas present in a higher elevations that were not present in the lowest elevation. Nestedness was defined as areas present in higher elevations that were also present in the lowest elevation.

Across our entire 7‐year dataset, there is consistency in where changes in the functional structure of assemblages take place. Functional turnover is largely seen through the introduction of species with traits indicating predatory specialization and life in open habitats (negative values on axes 1 and 2, Fig. 4). Functional nestedness results in extreme trait combinations being lost from the functional space. This leaves the assemblages at the highest elevations with species that possess generalized traits centred on the origin (0, 0) of the functional trait space. These species tend to be dietary generalists with no strong preference for open or closed environments (Figs 1, 4). These patterns could be reflecting deterministic community‐assembly processes. Environmental conditions may selectively filter which species are able to successfully establish and survive at each elevation. Such filtering would act on the functional trait values of the species. For this case, it would seem that species with extreme trait values are not able to exist at the highest elevations. This idea is consistent with the clustering and shrinking of phylogenetic diversity at higher elevations (Machac *et al*., 2011; Hoiss *et al*., 2012).

By standardizing our beta diversity measures against appropriate null models, however, we cannot support such a model of trait‐based community assembly. Functional beta diversity is random with respect to species beta diversity. In other words, our observed result is indistinguishable from a random assignment of trait values to species. This implies that the traits we investigate have no role in driving the elevational patterns of species beta diversity. We suggest that two factors combine to produce this ‘null’ result. First, there are fewer species at higher elevations than at low elevations (Bishop *et al*., 2014). Second, the structure of trait space (points in Fig. 1) and the species occurrence data (observed species beta diversity) are kept constant during each iteration of the null model. The combination of these factors produces a sampling effect whereby greater functional volumes are achieved with more species – giving rise to our patterns of functional turnover and nestedness. We cannot distinguish the observed patterns of functional beta diversity from this stochastic effect.

If the functional traits we use here are not responsible for the apparent deterministic species turnover, then what is? Factors such as soil structure and the presence of parasitoids are known to influence the structure of ant communities (LeBrun, 2005; Ríos‐Casanova *et al*., 2006). It seems unlikely that these would be able to drive the strong turnover of ant species we observe. There is no directional change in soil composition across the gradient. In addition, any top‐down forces that regulate ant species turnover would presumably have to change with elevation themselves. Instead, we suggest that currently unmeasured physiological traits such as thermal tolerances may be playing an important role in generating species turnover. Such traits have been suggested to be important for other terrestrial insects along elevational gradients (Hodkinson, 2005). Furthermore, the coexistence and distribution of ant species can be influenced by thermal regimes and species tolerances (Wittman *et al*., 2010; Wiescher *et al*., 2012).

An investigation into phylogenetic beta diversity (Leprieur *et al*., 2012) may yield further insights into the relative roles of deterministic and stochastic processes (e.g. Molina‐Venegas *et al*., 2015). This is particularly true if traits are distributed across the phylogeny in interesting ways, such as being clustered or segregated.

This study has begun to reveal in greater detail what kinds of ecological mechanisms may drive ant diversity across broad environmental gradients. We emphasize the importance of beta‐diversity partitioning and the functional‐trait view of ecology in fully appreciating the distribution and organization of biodiversity. Without using these techniques, we would have been unable to accurately describe how assemblages change with elevation, and we would not have been able to investigate whether a given set of ecologies were able to explain the changes in species distribution.

## **Biosketch**

**Tom R. Bishop** is a PhD student at the University of Liverpool. He is interested in understanding the structure of ecological communities and the distribution of biological diversity, particularly that of the ants.

Author contributions: T.R.B. and C.L.P. conceived the research questions. M.P.R. and B.J.v.R. designed and oversaw all historical data collection. T.R.B. collected morphological measurements. T.R.B. analysed the data and wrote the manuscript. All authors contributed to the final draft.

## Supporting information


**Appendix S1** Generalized linear mixed models using all pairwise combinations of elevational sites.
**Appendix S2** Plots showing the relationship between ant species and functional β_sor_ (total beta diversity), β_sim_ (turnover component) and β_sne_ (nestedness‐resultant component) and elevational distance.

## References

[jbi12537-bib-0001] Anderson, M.J. , Crist, T.O. , Chase, J.M. , Vellend, M. , Inouye, B.D. , Freestone, A.L. , Sanders, N.J. , Cornell, H.V. , Comita, L.S. , Davies, K.F. , Harrison, S.P. , Kraft, N.J.B. , Stegen, J.C. & Swenson, N.G. (2011) Navigating the multiple meanings of β diversity: a roadmap for the practicing ecologist. Ecology Letters, 14, 19–28. 21070562 10.1111/j.1461-0248.2010.01552.x

[jbi12537-bib-0002] Barton, K. (2013) MuMIn: multi‐model inference. R package version 1.9.13. http://cran.r-project.org/package=MuMIn.

[jbi12537-bib-0003] Baselga, A. (2010) Partitioning the turnover and nestedness components of beta diversity. Global Ecology and Biogeography, 19, 134–143.

[jbi12537-bib-0004] Baselga, A. & Orme, C.D.L. (2012) betapart: an R package for the study of beta diversity. Methods in Ecology and Evolution, 3, 808–812.

[jbi12537-bib-0005] Bates, D. , Maechler, M. , Bolker, B. , Walker, S. , Christensen, R.H.B. , Singmann, H. & Dai, B. (2014) lme4: linear mixed‐effects models using Eigen and S4. R package version 1.1‐6. http://cran.r-project.org/package=lme4.

[jbi12537-bib-0006] Beaudrot, L. , Rejmánek, M. & Marshall, A.J. (2013) Dispersal modes affect tropical forest assembly across trophic levels. Ecography, 36, 984–993.

[jbi12537-bib-0007] Bernadou, A. , Espadaler, X. , Le Goff, A. & Fourcassié, V. (2015) Ant organization along elevational gradients in a temperate ecosystem. Insectes Sociaux, 62, 59–71.

[jbi12537-bib-0008] Bihn, J.H. , Gebauer, G. & Brandl, R. (2010) Loss of functional diversity of ant assemblages in secondary tropical forests. Ecology, 91, 782–792. 20426336 10.1890/08-1276.1

[jbi12537-bib-0009] Bishop, T.R. , Robertson, M.P. , van Rensburg, B.J. & Parr, C.L. (2014) Elevation–diversity patterns through space and time: ant communities of the Maloti‐Drakensberg Mountains of southern Africa. Journal of Biogeography, 41, 2256–2268.

[jbi12537-bib-0010] Bond, W.J. , Smythe, K.‐A. & Balfour, D.A. (2001) *Acacia* species turnover in space and time in an African savanna. Journal of Biogeography, 28, 117–128.

[jbi12537-bib-0011] Brown, W.L. (1953) Revisionary studies in the ant tribe Dacetini. American Midland Naturalist, 50, 1–137.

[jbi12537-bib-0012] Brühl, C.A. , Mohamed, M. & Linsenmair, K.E. (1999) Altitudinal distribution of leaf litter ants along a transect in primary forests on Mount Kinabalu, Sabah, Malaysia. Journal of Tropical Ecology, 15, 265–277.

[jbi12537-bib-0013] Cardoso, P. , Rigal, F. , Carvalho, J.C. , Fortelius, M. , Borges, P.A.V. , Podani, J. & Schmera, D. (2014) Partitioning taxon, phylogenetic and functional beta diversity into replacement and richness difference components. Journal of Biogeography, 41, 749–761.

[jbi12537-bib-0014] Carvalho, J.C. , Cardoso, P. & Gomes, P. (2012) Determining the relative roles of species replacement and species richness differences in generating beta‐diversity patterns. Global Ecology and Biogeography, 21, 760–771.

[jbi12537-bib-0015] Chase, J.M. & Myers, J.A. (2011) Disentangling the importance of ecological niches from stochastic processes across scales. Philosophical Transactions of the Royal Society B: Biological Sciences, 366, 2351–2363. 10.1098/rstb.2011.0063 PMC3130433 21768151

[jbi12537-bib-0016] Fukami, T. , Bezemer, T.M. , Mortimer, S.R. & van der Putten, W.H. (2005) Species divergence and trait convergence in experimental plant community assembly. Ecology Letters, 8, 1283–1290.

[jbi12537-bib-0017] Gaston, K.J. & Blackburn, T.M. (2000) Pattern and process in macroecology. Blackwell Science, Oxford.

[jbi12537-bib-0018] Gibb, H. & Parr, C.L. (2013) Does structural complexity determine the morphology of assemblages? An experimental test on three continents. PLoS ONE, 8, e64005. 23691137 10.1371/journal.pone.0064005 PMC3656910

[jbi12537-bib-0019] Gotelli, N.J. (2000) Null model analysis of species co‐occurrence patterns. Ecology, 81, 2606–2621.

[jbi12537-bib-0020] Gronenberg, W. , Paul, J. , Just, S. & Hölldobler, B. (1997) Mandible muscle fibers in ants: fast or powerful? Cell and Tissue Research, 289, 347–361. 9211838 10.1007/s004410050882

[jbi12537-bib-0021] Harrison, S. , Ross, S.J. & Lawton, J.H. (1992) Beta diversity on geographic gradients in Britain. Journal of Animal Ecology, 61, 151–158.

[jbi12537-bib-0022] Hodkinson, I.D. (2005) Terrestrial insects along elevation gradients: species and community responses to altitude. Biological Reviews, 80, 489–513. 16094810 10.1017/s1464793105006767

[jbi12537-bib-0023] Hoiss, B. , Krauss, J. , Potts, S.G. , Roberts, S. & Steffan‐Dewenter, I. (2012) Altitude acts as an environmental filter on phylogenetic composition, traits and diversity in bee communities. Proceedings of the Royal Society B: Biological Sciences, 279, 4447–4456. 22933374 10.1098/rspb.2012.1581 PMC3479805

[jbi12537-bib-0024] Hölldobler, B. & Wilson, E.O. (1990) The ants. Springer, Berlin.

[jbi12537-bib-0025] Kraft, N.J.B. , Comita, L.S. , Chase, J.M. , Sanders, N.J. , Swenson, N.G. , Crist, T.O. , Stegen, J.C. , Vellend, M. , Boyle, B. , Anderson, M.J. , Cornell, H.V. , Davies, K.F. , Freestone, A.L. , Inouye, B.D. , Harrison, S.P. & Myers, J.A. (2011) Disentangling the drivers of β diversity along latitudinal and elevational gradients. Science, 333, 1755–1758. 21940897 10.1126/science.1208584

[jbi12537-bib-0026] Lamanna, C. , Blonder, B. , Violle, C. *et al* (2014) Functional trait space and the latitudinal diversity gradient. Proceedings of the National Academy of Sciences USA, 111, 13745–13750. 10.1073/pnas.1317722111 PMC4183280 25225365

[jbi12537-bib-0027] LeBrun, E.G. (2005) Who is the top dog in ant communities? Resources, parasitoids, and multiple competitive hierarchies. Oecologia, 142, 643–652. 15688219 10.1007/s00442-004-1763-4

[jbi12537-bib-0028] Leprieur, F. , Albouy, C. , Bortoli, D.J. & Cowman, P.F. (2012) Quantifying phylogenetic beta diversity: distinguishing between ‘true’ turnover of lineages and phylogenetic diversity gradients. PLoS ONE, 7, e42760. 22912736 10.1371/journal.pone.0042760 PMC3422232

[jbi12537-bib-0029] Lessard, J.‐P. , Dunn, R.R. , Parker, C.R. & Sanders, N.J. (2007) Rarity and diversity in forest ant assemblages of Great Smoky Mountains National Park. Southeastern Naturalist, 6, 215–228.

[jbi12537-bib-0030] Longino, J.T. & Colwell, R.K. (2011) Density compensation, species composition, and richness of ants on a neotropical elevational gradient. Ecosphere, 2, 29.

[jbi12537-bib-0031] Machac, A. , Janda, M. , Dunn, R.R. & Sanders, N.J. (2011) Elevational gradients in phylogenetic structure of ant communities reveal the interplay of biotic and abiotic constraints on diversity. Ecography, 34, 364–371.

[jbi12537-bib-0032] Marini, L. , Bertolli, A. , Bona, E. , Federici, G. , Martini, F. , Prosser, F. & Bommarco, R. (2013) Beta‐diversity patterns elucidate mechanisms of alien plant invasion in mountains. Global Ecology and Biogeography, 22, 450–460.

[jbi12537-bib-0033] McGill, B.J. , Enquist, B.J. , Weiher, E. & Westoby, M. (2006) Rebuilding community ecology from functional traits. Trends in Ecology and Evolution, 21, 178–185. 16701083 10.1016/j.tree.2006.02.002

[jbi12537-bib-0034] Molina‐Venegas, R. , Aparicio, A. , Slingsby, J.A. , Lavergne, S. & Arroyo, J. (2015) Investigating the evolutionary assembly of a Mediterranean biodiversity hotspot: deep phylogenetic signal in the distribution of eudicots across elevational belts. Journal of Biogeography, 42, 507–518.

[jbi12537-bib-0035] Mouchet, M.A. , Villéger, S. , Mason, N.W.H. & Mouillot, D. (2010) Functional diversity measures: an overview of their redundancy and their ability to discriminate community assembly rules. Functional Ecology, 24, 867–876.

[jbi12537-bib-0036] Mucina, L. & Rutherford, M.C. (2006) The vegetation of South Africa, Lesotho and Swaziland. South African National Biodiversity Institute, Pretoria.

[jbi12537-bib-0037] Nakagawa, S. & Schielzeth, H. (2013) A general and simple method for obtaining *R* ^2^ from generalized linear mixed‐effects models. Methods in Ecology and Evolution, 4, 133–142.

[jbi12537-bib-0038] Olivier, P.I. & van Aarde, R.J. (2014) Multi‐scale sampling boosts inferences from beta diversity patterns in coastal forests of South Africa. Journal of Biogeography, 41, 1428–1439.

[jbi12537-bib-0039] Paknia, O. & Pfeiffer, M. (2011) Hierarchical partitioning of ant diversity: implications for conservation of biogeographical diversity in arid and semi‐arid areas. Diversity and Distributions, 17, 122–131.

[jbi12537-bib-0040] R Core Team (2013) R: a language and environment for statistical computing. R Foundation for Statistical Computing, Vienna, Austria Available at: http://www.r-project.org/.

[jbi12537-bib-0041] Ricotta, C. & Burrascano, S. (2008) Beta diversity for functional ecology. Preslia, 80, 61–72.

[jbi12537-bib-0042] Ríos‐Casanova, L. , Valiente‐Banuet, A. & Rico‐Gray, V. (2006) Ant diversity and its relationship with vegetation and soil factors in an alluvial fan of the Tehuacán Valley, Mexico. Acta Oecologica, 29, 316–323.

[jbi12537-bib-0043] Robertson, H.G. (2002) Comparison of leaf litter ant communities in woodlands, lowland forests and montane forests of north‐eastern Tanzania. Biodiversity and Conservation, 11, 1637–1652.

[jbi12537-bib-0044] Schielzeth, H. (2010) Simple means to improve the interpretability of regression coefficients. Methods in Ecology and Evolution, 1, 103–113.

[jbi12537-bib-0045] Schmera, D. & Podani, J. (2011) Comments on separating components of beta diversity. Community Ecology, 12, 153–160.

[jbi12537-bib-0046] Silva, R.R. & Brandão, C.R.F. (2010) Morphological patterns and community organization in leaf‐litter ant assemblages. Ecological Monographs, 80, 107–124.

[jbi12537-bib-0047] Smith, M.A. , Hallwachs, W. & Janzen, D.H. (2014) Diversity and phylogenetic community structure of ants along a Costa Rican elevational gradient. Ecography, 37, 720–731.

[jbi12537-bib-0048] Stevens, R.D. , Cox, S.B. , Strauss, R.E. & Willig, M.R. (2003) Patterns of functional diversity across an extensive environmental gradient: vertebrate consumers, hidden treatments and latitudinal trends. Ecology Letters, 6, 1099–1108.

[jbi12537-bib-0049] Swenson, N.G. (2014) Functional and phylogenetic ecology in R. Springer, New York.

[jbi12537-bib-0050] Swenson, N.G. , Anglada‐Cordero, P. & Barone, J.A. (2011) Deterministic tropical tree community turnover: evidence from patterns of functional beta diversity along an elevational gradient. Proceedings of the Royal Society B: Biological Sciences, 278, 877–884. 20861048 10.1098/rspb.2010.1369 PMC3049044

[jbi12537-bib-0051] Swenson, N.G. , Stegen, J.C. , Davies, S.J. , Erickson, D.L. , Forero‐Montaña, J. , Hurlbert, A.H. , Kress, W.J. , Thompson, J. , Uriarte, M. , Wright, S.J. & Zimmerman, J.K. (2012) Temporal turnover in the composition of tropical tree communities: functional determinism and phylogenetic stochasticity. Ecology, 93, 490–499. 22624204 10.1890/11-1180.1

[jbi12537-bib-0052] Traniello, J.F.A. (1987) Comparative foraging ecology of north temperate ants: the role of worker size and cooperative foraging in prey selection. Insectes Sociaux, 34, 118–130.

[jbi12537-bib-0053] Tuomisto, H. (2010) A diversity of beta diversities: straightening up a concept gone awry. Part 1. Defining beta diversity as a function of alpha and gamma diversity. Ecography, 33, 2–22.

[jbi12537-bib-0054] Ulrich, W. , Almeida‐Neto, M. & Gotelli, N.J. (2009) A consumer's guide to nestedness analysis. Oikos, 118, 3–17.

[jbi12537-bib-0055] Villéger, S. , Mason, N.W.H. & Mouillot, D. (2008) New multidimensional functional diversity indices for a multifaceted framework in functional ecology. Ecology, 89, 2290–2301. 18724739 10.1890/07-1206.1

[jbi12537-bib-0056] Villéger, S. , Ramos Miranda, J. , Flores Hernandez, D. & Mouillot, D. (2012) Low functional β‐diversity despite high taxonomic β‐diversity among tropical estuarine fish communities. PLoS ONE, 7, e40679. 22792395 10.1371/journal.pone.0040679 PMC3392234

[jbi12537-bib-0057] Villéger, S. , Grenouillet, G. & Brosse, S. (2013) Decomposing functional β‐diversity reveals that low functional β‐diversity is driven by low functional turnover in European fish assemblages. Global Ecology and Biogeography, 22, 671–681.

[jbi12537-bib-0058] Wang, J. , Soininen, J. , Zhang, Y. , Wang, B. , Yang, X. & Shen, J. (2012) Patterns of elevational beta diversity in micro‐ and macroorganisms. Global Ecology and Biogeography, 21, 743–750.

[jbi12537-bib-0059] Weiser, M.D. & Kaspari, M. (2006) Ecological morphospace of New World ants. Ecological Entomology, 31, 131–142.

[jbi12537-bib-0060] Whittaker, R.H. (1960) Vegetation of the Siskiyou Mountains, Oregon and California. Ecological Monographs, 30, 279–338.

[jbi12537-bib-0061] Wiescher, P.T. , Pearce‐Duvet, J.M.C. & Feener, D.H. (2012) Assembling an ant community: species functional traits reflect environmental filtering. Oecologia, 169, 1063–1074. 22294027 10.1007/s00442-012-2262-7

[jbi12537-bib-0062] Williams, P.H. , de Klerk, H.M. & Crowe, T.M. (1999) Interpreting biogeographical boundaries among Afrotropical birds: spatial patterns in richness gradients and species replacement. Journal of Biogeography, 26, 459–474.

[jbi12537-bib-0063] Wittman, S.E. , Sanders, N.J. , Ellison, A.M. , Jules, E.S. , Ratchford, J.S. & Gotelli, N.J. (2010) Species interactions and thermal constraints on ant community structure. Oikos, 119, 551–559.

[jbi12537-bib-0064] Wright, D.H. & Reeves, J.H. (1992) On the meaning and measurement of nestedness of species assemblages. Oecologia, 92, 416–428. 10.1007/BF00317469 28312609

