# Supplementary material for: Contrasting species and functional beta diversity in montane ant assemblages
Source: J Biogeogr. 2015 May 16;42(9):1776–86. doi: 10.1111/jbi.12537 (PMC4979679; doi:10.1111/jbi.12537)

*Journal of Biogeography*

**Supporting Information**

**Contrasting species and functional beta diversity in montane ant communities**

Tom R. Bishop, Mark P. Robertson, Berndt J. van Rensburg and Catherine L. Parr

**Appendix S1** Summary of generalized linear mixed models (GLMMs) explaining variation in observed and standardized beta diversity of ant communities within the Sani Pass, southern Africa. Modelling took place as described in the main text, except that all pairwise comparisons between elevations were included.

**Table S1** Model summaries for generalized linear mixed models explaining variation in observed and standardized beta diversity of ant communities within the Sani Pass, southern Africa. The best model, according to the bias-corrected Akaike information criterion (AIC_c_) is reported. β_sor_ is total beta diversity, β_sim_ is turnover and β_sne_ is nestedness. Marginal *R*^2^ (*R^2^*_m_), measuring variation explained by fixed effects only, and conditional *R*^2^ (*R*^2^_c_), measuring variation explained by both fixed and random effects, are given.

| Model summaries | β_sor_ | β_sim_ | β_sne_ |
| --- | --- | --- | --- |
| AIC_c_ | 10462.72 | 11135.52 | 10960.81 |
| *R*^2^_m_ | 0.20 | 0.15 | 0.15 |
| *R*^2^_c_ | 0.20 | 0.16 | 0.15 |

**Appendix S2** Plots showing the relationship between ant species and functional (a) β_sor_ (total beta diversity), (b) β_sim_ (turnover component) and (c) β_sne_ (nestedness-resultant component) and elevational distance in the Sani Pass, southern Africa. Red lines and circles indicate species beta diversity. Blue lines and triangles indicate functional beta diversity. Filled shapes and solid lines indicate data and mixed-model predictions for the wet season. Empty shapes and dotted lines indicate those for the dry season. Data points represent all pairwise comparisons between elevations. Data from all years in the dataset are modelled and plotted.


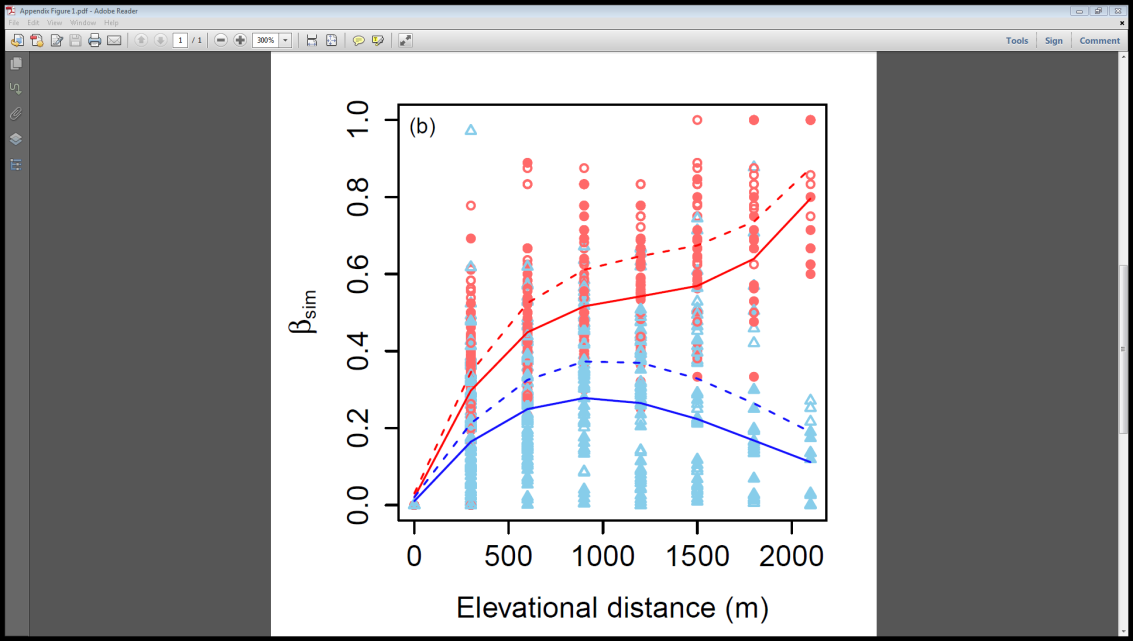

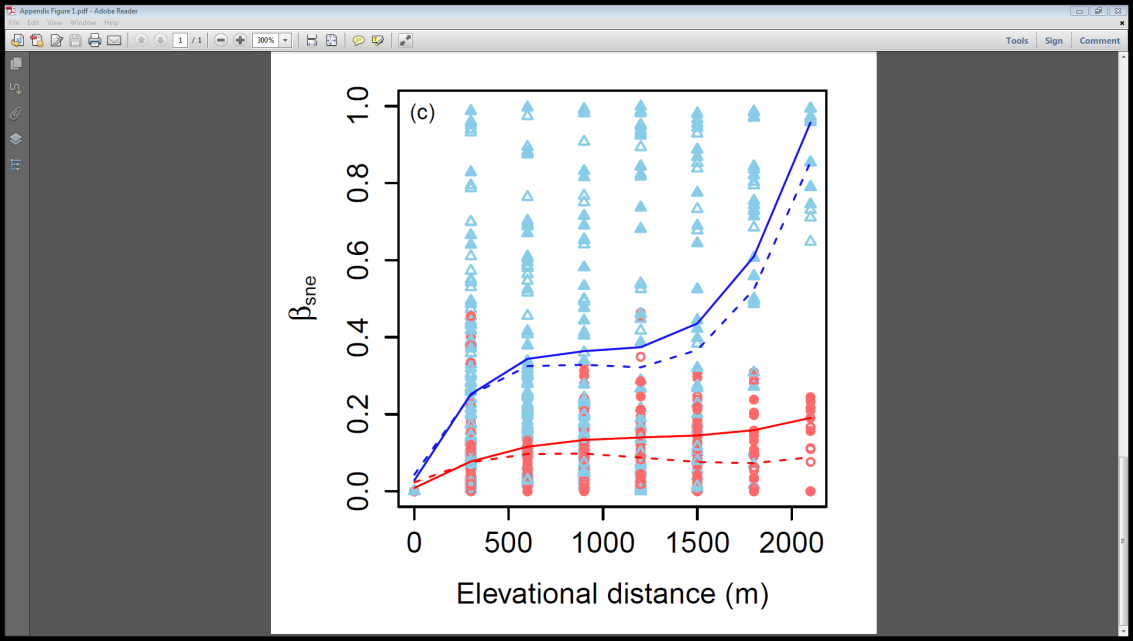

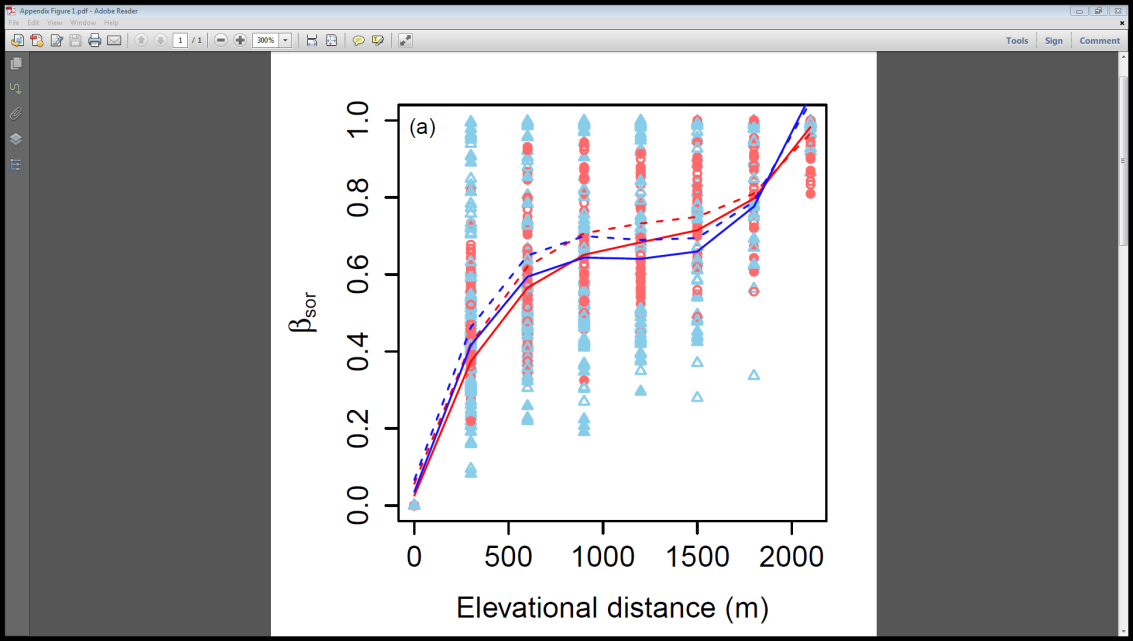

Supplement: Supplementary file 1 — Appendix S1 Generalized linear mixed models using all pairwise combinations of elevational sites. Appendix S2 Plots showing the relationship between ant species and functional βsor (total beta diversity), βsim (turnover component) and βsne (nestedness‐resultant component) and elevational distance. [file JBI-42-1776-s001.doc]
